# Machine learning and bioinformatic analyses link the cell surface receptor transcript levels to the drug response of breast cancer cells and drug off-target effects

**DOI:** 10.1371/journal.pone.0296511

**Published:** 2024-02-02

**Authors:** Musalula Sinkala, Krupa Naran, Dharanidharan Ramamurthy, Neelakshi Mungra, Kevin Dzobo, Darren Martin, Stefan Barth

**Affiliations:** 1 Department of Biomedical Sciences, School of Health Sciences, University of Zambia, Lusaka, Zambia; 2 Faculty of Health Sciences, Institute of Infectious Disease and Molecular Medicine & Department of Integrative Biomedical Sciences, Computational Biology Division, University of Cape Town, Cape Town, South Africa; 3 Faculty of Health Sciences, Institute of Infectious Disease and Molecular Medicine, Medical Biotechnology & Immunotherapy Research Unit, University of Cape Town, Cape Town, South Africa; 4 Faculty of Health Sciences, Department of Medicine, Division of Dermatology, Medical Research Council-SA Wound Healing Unit, Hair and Skin Research Laboratory, Groote Schuur Hospital, University of Cape Town, Anzio Road, Observatory, Cape Town, South Africa; 5 Faculty of Health Sciences, Department of Integrative Biomedical Sciences, South African Research Chair in Cancer Biotechnology, University of Cape Town, Cape Town, South Africa; Institute for Basic Science, REPUBLIC OF KOREA

## Abstract

Breast cancer responds variably to anticancer therapies, often leading to significant off-target effects. This study proposes that the variability in tumour responses and drug-induced adverse events is linked to the transcriptional profiles of cell surface receptors (CSRs) in breast tumours and normal tissues. We analysed multiple datasets to compare CSR expression in breast tumours with that in non-cancerous human tissues. Our findings correlate the drug responses of breast cancer cell lines with the expression levels of their targeted CSRs. Notably, we identified distinct differences in CSR expression between primary breast tumour subtypes and corresponding cell lines, which may influence drug response predictions. Additionally, we used clinical trial data to uncover associations between CSR gene expression in healthy tissues and the incidence of adverse drug reactions. This integrative approach facilitates the selection of optimal CSR targets for therapy, leveraging cell line dose-responses, CSR expression in normal tissues, and patient adverse event profiles.

## Introduction

The aberrant overexpression of cell surface receptors (CSRs) distinguishes cancer cells from their normal counterparts and is implicated in oncogenesis [1–4]. CSRs, encompassing receptor tyrosine kinases and G-protein coupled receptors, mediate extracellular and intracellular signalling interactions, often becoming dysregulated in cancer [5–7]. This dysregulation [8], along with their accessibility on the cell surface, renders CSRs prime targets for anticancer therapeutics [7,9–11]. Changes in CSR expression during oncogenesis may involve mutations, gene amplifications, or transcriptional modifications [12–14].

Traditional methods of selecting CSR targets for therapies, particularly in breast cancer, have focused on their overexpression or mutational alterations in tumours relative to adjacent non-cancerous tissues [15–17]. However, the expression profiles of CSRs in non-target organs, which could be affected by anticancer treatments, have been largely overlooked [15–17]. This oversight can lead to unintended effects on other organs when targeting CSRs in tumour-specific tissues.

The success of clinical trials for CSR-targeted treatments is often hampered by dose-limiting toxicities and unforeseen side effects [18–20]. Many of these adverse side effects likely stem from the drugs or therapeutic antibodies interacting with CSRs in non-cancerous tissues. [21,22]. An effective strategic approach to improve treatment success rates and reduce off-target toxicity might involve identifying CSRs that are upregulated in tumour cells but not in non-cancerous tissues, potentially reducing off-target toxicity.

In this study, we explore CSRs as therapeutic targets for breast cancer, aiming to reduce adverse effects by carefully selecting the CSRs. We mine data from various public databases and employ statistical techniques, machine learning, and predictive modelling to analyse the potential off-target toxicities of CSR-targeted drugs, including those with documented toxic effects. Our methodology is predicated on assumptions regarding the interplay between drug response, off-target toxicity, and the transcriptional profiles of breast cancer cells and normal tissues, which we evaluate against extensive high-quality laboratory data.

We employ a bioinformatics approach to dissect the relationship between CSR expression levels in breast cancer cell lines and their response to drugs targeting these receptors. Additionally, we assess the link between the adverse effects of CSR-directed drugs in treating breast tumours and the expression patterns of these CSRs in an array of normal tissues. Our computational analyses unveil the association between drug effects and CSR expression in breast cancer, offering insights that could extend to other cancers and improve the selection criteria for drug targets.

## Method

### Analysing CSR expression and drug response in breast cancer

**Fig 1:**
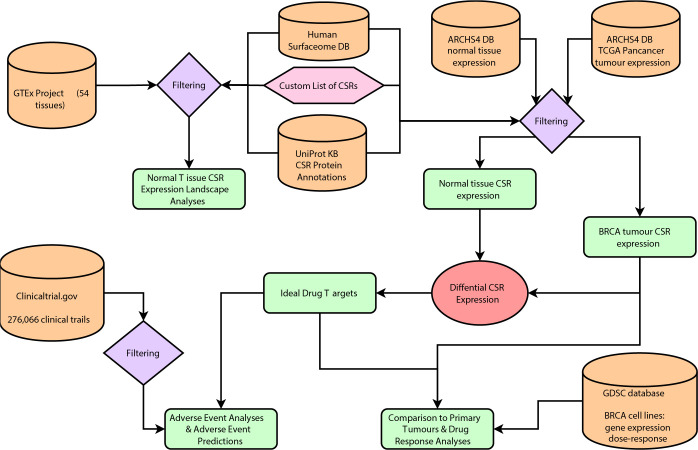
Graphical representation of the overall study method and computational analysis procedure.

To explore the link between cell surface receptor (CSR) expression and drug response in breast cancer, we accessed healthy tissue transcriptome profiles from the Genotype-Tissue Expression (GTEx) consortium (https://gtexportal.org/home/). Specifically, we extracted transcript abundance data for 54 different healthy tissues [23,24], as detailed in S1 File. A comprehensive list of CSRs was compiled using information from various sources, including academic literature, the UniProt Knowledgebase [25], the Surfaceome database [26], and the Gene Ontology Consortium [27] focusing on the Gene Ontology term “plasma membrane” (S1 File). This list facilitated the extraction of mRNA transcription data for genes identified as CSRs (see Fig 1). We then employed unsupervised hierarchical clustering on this data to analyse the expression patterns of CSRs across healthy tissues, visualising the results in a dendrogram.

### mRNA expression of CSR in breast tumours and healthy tissues

To characterise CSR expression across both healthy and breast cancer tissues, we utilised mRNA expression data from 9,685 individuals representing 54 healthy tissues, compiled by the GTEx consortium, and 1,079 breast cancer samples from The Cancer Genome Atlas (TCGA), accessed through the ARCHS4 database [28,29]. These datasets were processed uniformly using the reCount2 project’s computational pipeline [30], ensuring direct comparability. We applied t-Distributed Stochastic Neighbourhood Embedding [31] to identify clustering patterns between breast cancer tumours and healthy tissues, aiming to discern the distinct expression landscapes of CSRs in these different tissue types.

### Identifying optimal CSR target for anticancer drugs

Our hypothesis posits that anticancer drugs targeting CSRs may also induce off-target adverse effects due to CSR expression in healthy tissues. Thus, in this study, we defined the most suitable drug target CSRs as those upregulated in disease states compared to all healthy tissues. To identify these CSRs, we conducted differential gene expression analysis using the negative binomial test [32]. This involved comparing CSR transcript levels between breast tumour mRNA data from TCGA and each healthy tissue type from the GTEx project (see S2 File). CSRs upregulated (adjusted p-value < 0.05 and fold change > 2) across all comparisons between breast cancer and healthy tissues were identified as "ideal" drug and antibody targets.

### Predicting breast cancer subtypes based on CSR transcription data

Given that breast tumours are classified using the PAM50 scheme (Luminal A, Luminal B, Normal-like, Basal, and HER-2 positive), our goal was to replicate this subtyping using only CSR mRNA transcripts. Firstly, we utilised the TCGA-provided PAM50 subtypes and CSR gene transcript levels for each breast cancer sample. Then, we employed an embedded feature selection method based on a boosted decision tree-based machine learning algorithm, identifying 50 CSR features crucial for predicting PAM50 subtypes [33,34]. Subsequently, we developed an ensemble prediction model aggregating 20 decision trees [35] using Random Under Sampling Boosting [36]. This model was then used to classify the TCGA breast tumours into Luminal A, Luminal B, Normal-like, Basal, and HER-2 positive subtypes.

### Evaluating the impact of CSR transcription on the drug response of PAM50 cancer subtypes

Our aim was to explore the potential link between CSR mRNA transcription and the response of breast cancer cell lines, representing various PAM50 subtypes, to anticancer drugs. We used dose-response data from the Genomics of Drug Sensitivity in Cancer (GDSC) database [37] focusing on breast cancer cell lines previously classified into four distinct PAM50 subtypes by Dai et al. [38].

We performed pairwise comparisons of drug responses among different PAM50 subtype cell line groups using the Student’s t-test with unequal variance assumption for each of the 32 anticancer drugs studied (refer to S4 File).

### Comparison of CSR transcription in primary tumours and cancer cell lines

To compare CSR mRNA transcription between primary breast cancer PAM50 subtypes in the TCGA database and their corresponding PAM50 cancer cell lines in the GDSC database, we analysed differentially expressed transcripts. First, we used the Welch test to identify differentially expressed CSR transcripts between each pair of PAM50 subtypes within the GDSC cancer cell lines. Subsequently, we compared the list of differentially expressed CSRs between each pair of breast cancer PAM50 subtypes (e.g., Basal vs HER2 positive) in primary tumours with those in the corresponding cancer cell lines (e.g., Basal vs HER positive; see S3 File). We anticipated finding a concordant pattern of upregulated and downregulated CSRs in each matched comparison between the primary tumours and cancer cell lines.

### Correlating CSR transcription with drug response in breast cancer cell lines

To investigate the relationship between CSR transcription and drug response, we categorised breast cancer cell lines into two groups for each drug-response comparison, irrespective of their PAM50 subtype classification: 1) cell lines overexpressing the CSR target of the drug, and 2) those underexpressing the target CSR. For this classification, we retrieved the transcription profiles of the drug targets across cell lines from the GDSC and applied z-normalisation to these profiles [39]. Using a threshold of one standard deviation, cell lines with a z-score above 1 were classified as having higher expression of the drug target, while those with a z-score below -1 were considered as having lower target expression. Cell lines with z-scores ranging from -1 to 1 were excluded from the analysis for each specific drug.

To evaluate the differences in drug responses between these two groups (i.e., cell lines with high versus low drug target expression), we utilised the Welch test. This analysis was performed on the area-under-the-curve (AUC) values from the dose-response curves of each cell line group, thereby comparing their mean drug responses (refer to S4 File). This approach aimed to elucidate the impact of differential CSR expression levels on the efficacy of drug treatments in breast cancer cell lines.

### Validation of the ideal targets on reported adverse events

We accessed data from https://clinicaltrials.gov/ of breast cancer clinical trials that administered a single anticancer drug or antibody [40]. Further, we retrieved the actual targets of these drugs from the Pharos database (https://pharos.nih.gov [41]) and the Drug Gene Interaction Database (http://dgidb.org [42]), focusing on trials that used drugs targeting CSRs. The clinical trial records provided information on participants, including treatments and adverse events experienced (see S5 File). Finally, we collated data on anticancer treatments and corresponding adverse events for each drug.

Clinical trials were divided into two categories: those using CSRs identified as "ideal" targets (highly expressed in breast cancer compared to other healthy tissues) and those using "other targets". Trials involving "ideal targets" reported adverse events for 544 individuals, whereas those involving "other targets" reported events for 501 individuals. We compared the reported proportions and numbers of individuals experiencing adverse events between these two clinical trial groups.

### Predicting adverse events using CSR transcription levels in healthy tissues

We associated reported adverse events in clinical trials with specific body tissues, categorising events like "Skin and subcutaneous tissue disorders" to the skin and "cardiac disorders" to the heart (see S5 File). Each adverse event was linked to the expression levels of CSR transcripts in the corresponding healthy tissue, as obtained from GTEx data. For each drug used in the clinical trials, we sourced the drug target from Pharos and Drug Gene Interaction databases, focusing on trials using drugs targeting CSRs [41,42].

We trained a machine learning model to predict adverse events in various healthy tissues, using the tissue-specific adverse events and CSR transcript levels of the drug target. Specifically, we trained 20 different machine learning regression models, including linear regression (using a simple linear model, interaction terms, and stepwise methods), decision trees regression (of various tree and leaf sizes), support vector machines regression (of various kernel scales, kernel functions, and box constraints), ensemble trees (boosted and bagged trees), and Gaussian process regression (of various kernel scales, kernel functions, signal standard deviation, and sigma).

The two best-performing models, based on 5-fold cross-validation accuracy, were the quadratic support vector machines model [43] (root mean squared error = 0.042) and the squared exponential Gaussian process regression [44] (root mean squared error = 0.043). We combined these two best-performing models by training an ensemble machine learning algorithm based on quadratic support vector machine regression and squared exponential Gaussian process regression. We used this ensemble model to predict adverse events for each anticancer drug, based on the CSR expression in the target tissues.

### Statistical analysis and data visualisation

All statistical analyses were conducted using MATLAB version 2020b [45]. For categorical variable associations, Fisher’s exact test was used. The independent sample Student t-test, Welch test, and one-way analysis of variance were employed for comparing continuous variables. Single comparisons were deemed significant at p < 0.05, and for multiple comparisons, p-values were adjusted using the Benjamini-Hochberg method [46]. All results and data were visualised using MATLAB and Tableau version 2019.1.7 [47].

## Results

### The transcriptional landscape of CSRs across breast tumours and healthy tissues

**Fig 2:**
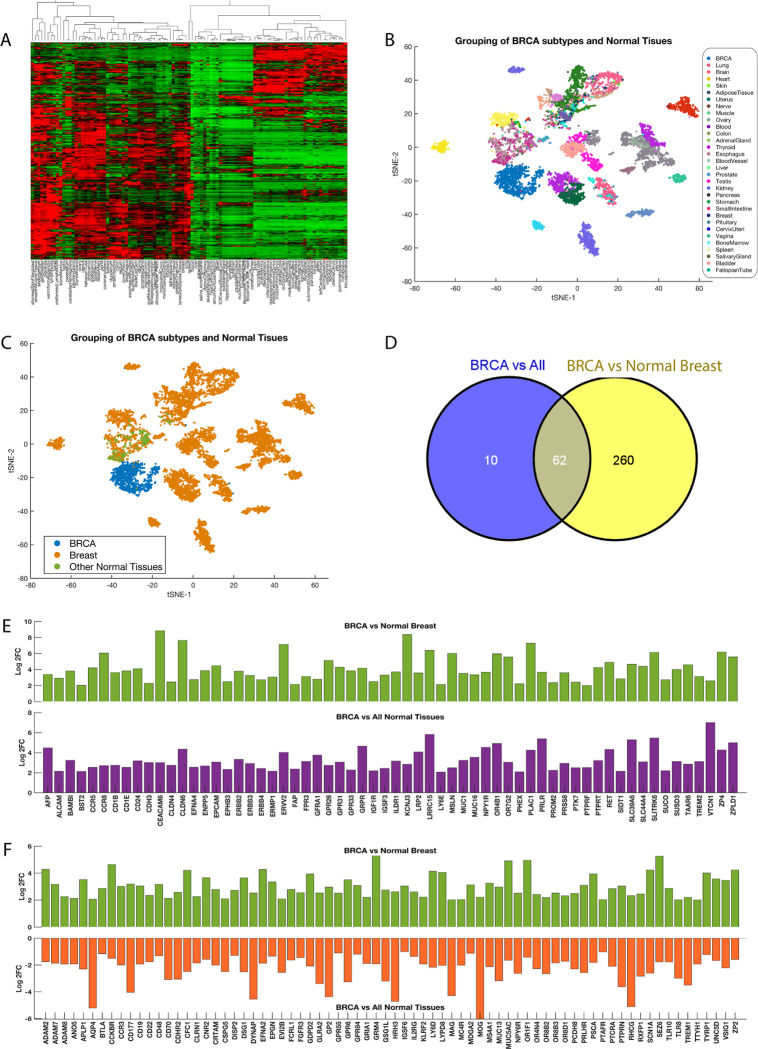
Comprehensive analysis of CSR transcription in breast cancer and normal tissues. (A) Heatmap of 54 normal human tissues displaying mRNA transcription data of CSRs, produced through unsupervised hierarchical clustering with the Euclidean distance metric, row standardisation, and complete linkage. (B) Clustering of 1,320 breast cancer samples (represented as blue points) and normal tissue samples (represented as points in various colours) based on mRNA transcript levels of CSRs. The samples are coloured based on the tissue type from which the measured CSR transcripts were obtained. (C) Clustering of 1,320 breast cancer samples (represented as blue points), 13,390 normal breast tissue samples (represented as green points), and 34,000 other normal tissue samples (represented as orange points) based on mRNA transcript levels of CSRs. t-SNE was employed for sample clustering visualization with the exact algorithm and standardised Euclidean distance metric. (D) Comparison of the number of CSR transcripts upregulated between breast tumours and all other normal tissues and breast tumours and normal breast showing that 62 transcripts are commonly upregulated between the two sets of comparisons. Refer to supplementary files 1 and 2 for complete results of the differentially expressed CSR transcripts. (E) Bar graph depicting CSR transcripts that are commonly upregulated for two comparisons: 1) between breast tumours and normal breast and 2) between breast tumours and all other healthy tissues. Labels in the lower panel (2) also apply to the upper panel (1). (F) Representation of CSR transcripts that demonstrate a reverse signature, meaning they are upregulated in breast tumours compared to the healthy breast but downregulated in breast tumours compared to all other normal tissues. Labels in the lower panel apply to the upper panel as well.

In our quest to discern CSR transcription patterns, we analysed mRNA expression data from 54 organs and tissues sourced from the GTEx project [23,24,48] focusing on 1,140 CSR-encoding genes. Unsupervised hierarchical clustering was employed to examine CSR expression variations across these healthy tissues (Fig 2A).

We then compared mRNA expression data from 1,091 breast cancer samples from the TCGA [29] with CSR mRNA expression data from 9,658 healthy samples, including 218 from healthy breast tissue, also derived from the GTEx project (Fig 2B and 2C).

Our analysis identified 634 and 581 CSR transcripts differentially expressed (adjusted p-value < 0.05 with a fold-change > 2 or < -2) between breast tumours and healthy breast tissue, and between breast tumours and other healthy tissues, respectively. Notably, 322 CSR transcripts were significantly elevated in breast tumours compared to healthy breast tissue, with *CEACAM6* (log2FC = 8.8), KCNJ (8.4), and *CLDN6* (7.6) among the most upregulated (Fig 2D and S1 File). Furthermore, 72 CSRs showed higher expression in breast tumours versus non-breast healthy tissues, including *VTCN1* (log2FC = 7.0), LRRC (5.8), and *SLITRK6* (5.5) (Fig 2D). Only 62 transcripts were commonly upregulated in breast tumours against both healthy breast and non-breast tissues, featuring *ERBB2*, *ERBB3*, *EPCAM*, and *IGFR* (Fig 2E).

Conversely, of the 511 CSRs overexpressed in breast tumours relative to healthy breast tissue, 72 were significantly underexpressed when compared to non-breast healthy tissues, including well-established drug targets like *FGFR3*, *CD48*, and *CCR3* (Fig 2F). This finding suggests that a higher expression of target CSRs in breast tumours versus healthy breast tissue does not guarantee similar expression levels when compared to other healthy tissues.

The natural variation in CSR expression among tissues underscores the complexity in developing CSR-targeting anticancer drugs. The prevalence of targeted CSRs in non-cancerous tissues is likely a principal contributor to the dose-limiting toxicity commonly observed with CSR-targeted therapies [21,22].

### Selecting optimal CSR targets to minimise off-target toxicity

**Fig 3:**
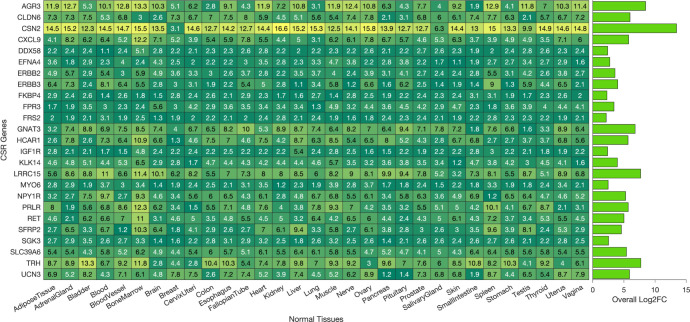
Upregulated CSR transcripts in breast tumors: Log-2 fold-change heatmap. The heatmap of the log-2 fold-change values of the CSR transcripts that are consistently upregulated in our comparison of breast tumours against every other healthy tissue.

We hypothesized that CSRs with higher expression levels on cancer cells relative to all healthy tissues could mitigate the off-target toxic effects seen with CSR-targeted anticancer treatments. In prioritising CSRs for targeting, we explored whether a threshold of mRNA expression could be established. Our approach accounted for expression across all tissues, aiming to circumvent essential tissues—such as the brain, heart, lungs, liver, and kidneys—while minimising toxicities in less critical tissues (refer to the methods section). An algorithm was employed to single out CSRs markedly overexpressed in breast tumours versus any healthy tissue. This comparative analysis across healthy tissues identified 26 CSRs with substantially higher expression in breast tumours, suggesting they are viable targets for reducing off-target cytotoxicity (Fig 3; see also S1 and S2 Files). Among the identified CSRs, several have been extensively studied and validated as significant in breast cancer pathogenesis and therapy. Notably, ERBB2, also known as HER2, has been well-documented for its overexpression in breast cancer and is a validated therapeutic target [49,50], as is EGFR, which is implicated in various cancers including breast cancer [51,52]. Other notable CSRs such as IGF1R [53], FGFR2 [54], and RET [55] have also been extensively studied, with evidence supporting their roles in tumourigenesis and as potential therapeutic targets.

### Divergent CSR transcription profiles in breast cancer subtypes and implications for classification

**Fig 4:**
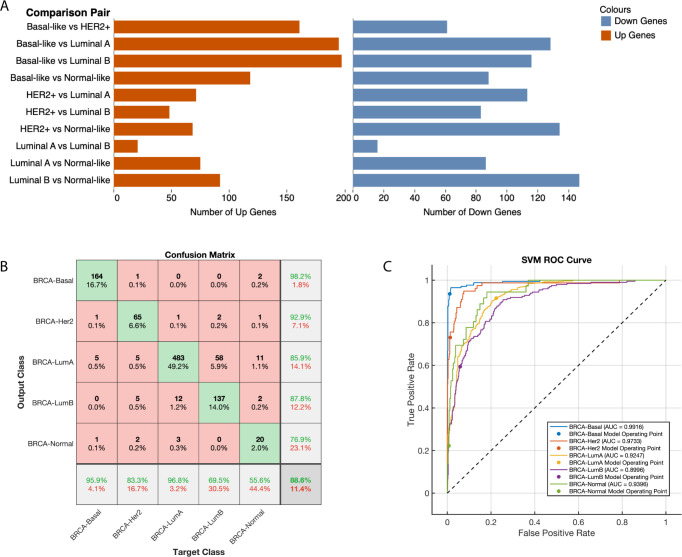
Comparative analysis and predictive performance of CSR transcripts in PAM50 breast cancer subtypes. **(A)** Bar graphs showing the number of differentially expressed CSR transcripts between each pairwise comparison of the PAM50 breast cancer subtype (x-axis). The two bar graphs are plotted for the upregulated transcripts between each comparison (left) and downregulated transcripts between each comparison (right; also see S3 File). **(B)** A plot of the confusion matrix. The diagonal cells correspond to correctly classified observations (green cells). The off-diagonal cells correspond to incorrectly classified observations (red cells). The number of observations and the percentage of the total observations are shown in each cell. The column to the far right shows the precision (or positive predictive value rate) in green text and the false discover rate shown in red text. The row at the bottom of the plot shows the recall (or true-positive rate) in green text and the false-negative rate in red. The cell in the bottom right of the plot shows the overall accuracy. **(C)** shows the ROC-AUC (Receiver Operated Characteristic-Area Under the Curve) for the PAM50 breast cancer classes. The ROC curves show the true-positive rate versus the false-positive rate for predictions of the classes made by the trained classifier. The coloured markers on the plots show the values of the false positive rate and the true-positive rate of the trained classifier toward predicting the PAM50 subtype of breast cancer using CSRs.

Breast tumours are categorised into five molecular subtypes—Luminal A, Luminal B, Normal-like, Basal-like, and HER-2 positive—based on the PAM50 gene signature [33,34]. Our comparative analysis of CSR transcript levels across these subtypes, as designated in the TCGA, revealed pronounced differences in CSR gene expression (Fig 4A and S3 File). The greatest number (323 differentially expressed transcripts) was between Basal-like and Luminal A tumours, while the smallest difference (32 transcripts) was noted between Luminal A and Luminal B tumours. These findings highlight a distinct transcriptional divergence between Basal-like tumours and Luminal A and B subtypes, which have more analogous transcriptional profiles.

Given the pivotal role of PAM50 subtyping in breast cancer management and prognosis, we investigated if variations in CSR mRNA levels could alone accurately subtype breast tumours. Utilising the TCGA’s PAM50 annotations and CSR transcription data, we developed a supervised machine learning model for classification (see methods section).

Employing an ensemble-boosted decision tree model yielded a high predictive accuracy for PAM50 subtypes (average area under the receiver operating characteristic curve of 93% and classification accuracy of 89%), based solely on the levels of 1,140 CSR transcripts (Fig 3B and 3C). The model demonstrated positive predictive values of 85.9% for Luminal A, 87.8% for Luminal B, 76.9% for Normal-like, 98.2% for Basal-like, and 92.9% for HER-2 positive tumours (Fig 4). Collectively, these results indicate that HER-2 and Basal-like tumours possess the most distinct CSR transcription profiles, with Luminal A and B subtypes being less distinguishable.

### Drug response correlation with CSR expression in breast cancer cell lines and PAM50 subtypes

We examined the variance in drug response profiles of breast cancer cell lines to CSR-targeting therapeutics, in correlation with their PAM50 subtype classification. Utilising the Genomics of Drug Sensitivity in Cancer (GDSC) database [37] we analysed drug responses of Luminal A, Luminal B, Basal-like, and HER2 cell lines to thirteen CSR-targeted drugs. Pairwise comparisons revealed no significant differences in drug response profiles across the subtypes, except for Basal-like versus Luminal A (adjusted p-value = 0.0079), post-multiple comparison correction (see S1 Fig and S4 File).

We posited that CSR transcription profiles in the GDSC database’s breast cancer cell lines might diverge from those in primary tumours, such as those catalogued in the TCGA database. Consequently, this variance could influence the cell lines’ drug responses, contingent on the expression levels of the CSR targeted by the drug. To verify this hypothesis, we concentrated our analysis on CSR transcripts that showed consistent differential expression between cell lines categorised by distinct PAM50 subtypes.

**Fig 5:**
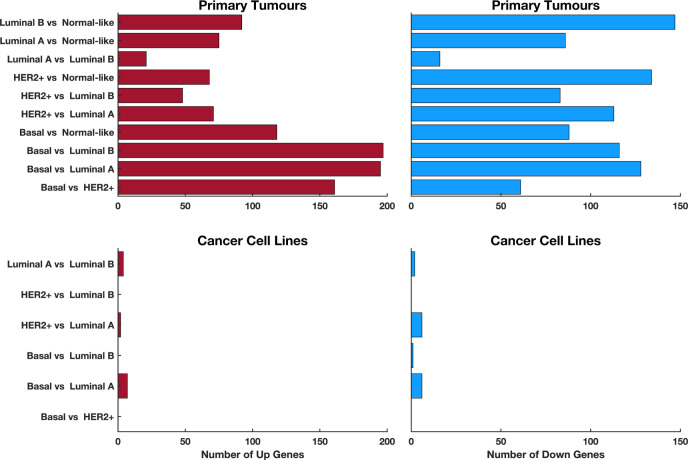
Comparison of the differentially expressed CSR transcripts between each pair of PAM50 breast cancer subtypes. The two plots show the comparison made for the TCGA primary tumours, whereas the two bottom plots show the comparison for the GDSC breast cancer cell lines. The plots on the left column show the upregulated transcripts between each comparison, and those on the right show the downregulated transcripts between each comparison.

We observed no significant differential expression (adjusted p-values < 0.05 and a two-fold change) of CSR transcripts between Basal-like and HER2+ subtypes, or between HER2+ and Luminal B subtypes. Only one CSR transcript exhibited significant variance between Basal-like and Luminal B cell lines, starkly contrasting with the substantial differences in CSR expression in primary tumours across PAM50 subtypes (Fig 5; andS4 File). This finding implies that breast cancer cell lines display a more homogenised pattern of CSR expression across subtypes compared to primary tumours, potentially explaining the uniform drug response profiles seen across different PAM50 subtype cell lines.

**Fig 6:**
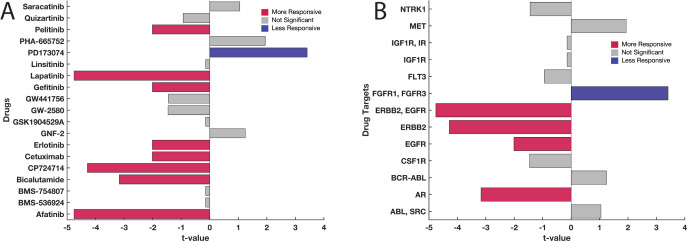
Comparing drug response to CSR-targeting drugs in cell lines with varying transcript levels. Each bar indicates the t-value calculated using the Welch test. The bars are coloured based on the level of statistical significance. Red bars denote statistically significant (p-value < 0.05) increased response to the drug for the cell lines overexpressing the targets compared to those under-expressing the target. Grey bars denote no statistically significant difference in the drug response. Blue bars denote statistically significant lowered response to the drug. **(A)** The comparisons were made across each drug represented in the GDSC database. **(B)** the comparisons are made across drugs that are grouped based on their target CSRs. For the test statistics relating to each comparison, refer to supplementary file 4.

Hence, we shifted our focus from PAM50 classification to the specific CSR expression levels when assessing responses to CSR-targeted drugs in cell lines. We categorised the cell lines into higher or lower CSR expression groups for each drug-response assay (refer to the Methods section). Remarkably, significant variances in drug responses were observed in 42% (8 of 19) of the examined anticancer drugs between the two expression groups (Fig 6 and S4 File). Furthermore, 15 out of 19 drugs demonstrated negative t-values, suggesting enhanced efficacy in cell lines with higher CSR expression. These results imply a linkage between the transcription levels of targeted CSRs and the therapeutic effectiveness of most evaluated anticancer drugs.

### Correlation between adverse drug reactions and CSR expression of in healthy tissues

We proposed that the off-target toxicity of drugs targeting CSRs is linked to the transcriptional levels of these CSR genes in healthy tissues. To explore this, we reviewed 224 clinical drug trials for breast cancer, extracting data on the anticancer drugs tested and their associated adverse events, specifically focusing on body tissues with available CSR transcription data (refer to the Methods section and S5 File).

We categorised the trials into two groups: (1) trials involving drugs targeting CSRs with higher expression in breast tumours than in healthy tissues, termed "ideal targets" (49 trials [22%], encompassing 10,191 participants); and (2) trials targeting other CSRs, labelled "other targets" (175 trials [78%], with 40,949 participants). We then compared the incidence of adverse events between these groups.

**Fig 7:**
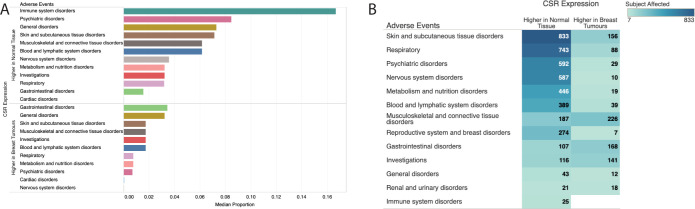
Adverse event distribution in breast cancer trials for targeted and non-targeted CSR drugs. **(A)** Distribution of adverse events reported in the clinical trial of breast cancer for drugs that target specific CSRs. The group are segregated by the types of drugs that are used to treat breast cancer patients: (1) Those that target highly expressed CSRs (the ideal targets) in breast tumours and (2) those that do not. The median proportion of individuals who experienced adverse events is reported for each bar graph. The colour shows details about adverse events. **(B)** Percentage of the total count of each category for each reported adverse in clinical trials that utilised the anticancer drug filgrastim, which targets CSF3R.

Our analysis indicated that trials involving "ideal targets" reported significantly fewer drug-related adverse events compared to the "other targets" trials (Chi-square test, χ2 = 15.2, p-value = 9.8 x 10^−5^; Fig 7). Specifically, the median rate of adverse effects in the "other targets" trials was 4.8%, markedly higher than the 1.9% in "ideal targets" trials (rank-sum test statistic = 186.5; p-value = 0.0094), across diverse adverse effect categories (Fig 7). In the 49 "ideal target" trials, 12 unique anticancer drugs were used, including lapatinib (targeting ERBB2), afatinib (ERBB2 and ERBB4), trastuzumab (ERBB2), and cabozantinib (RET). Conversely, the 175 "other target" trials employed 74 different drugs, such as filgrastim (targeting CSF3R), etelcalcetide (CASR), and bendamustine (CD69) (see S2 Fig).

This data suggests that drugs targeting "other targets" may induce more adverse effects due to their elevated expression in non-tumour tissues. For example, CSF3R, among the "other targets," is 338-fold more expressed in blood cells than in breast tumours (adjusted p-value < 1 x 10^−350^). Analysis of three clinical trials using filgrastim (targeting CSR3R) revealed a high incidence of blood and lymphatic system disorders, representing 17(53%) of the 32 different adverse events reported (Fig 7B). Notably, in trial NCT02104830, anaemia was observed in 44% of participants, leukopenia in 95%, and neutropenia in 95% (S3 Fig).

In conclusion, our results indicate a strong association between adverse reactions to CSR-targeted drugs and the expression levels of these CSRs in healthy tissues.

### Predicting adverse drug toxicity in healthy tissues using machine learning

We utilised machine learning methods to ascertain if clinical trial data could predict adverse drug toxicity events in healthy tissues. This involved extracting tissue-level mRNA transcript data for CSRs targeted in clinical trials reporting adverse events in healthy tissues (refer to the Methods section). We trained an ensemble machine learning model, incorporating Gaussian process regression [44] and support vector machines [43] and evaluated the model’s performance in predicting drug-induced adverse effects using an independent test dataset (see Methods section).

**Fig 8:**
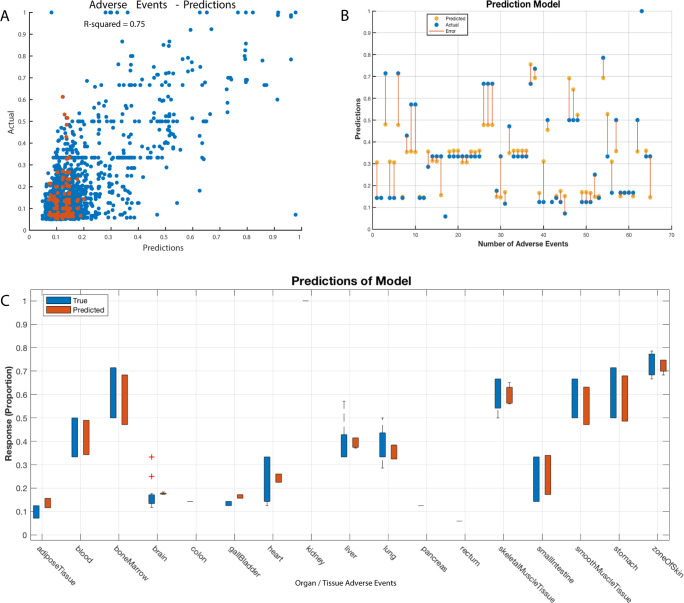
Model predictions vs. actual responses in drug adverse event profiling. **(A)** A scatter plot showing the predicted response (y-axis) of the machine learning model plotted against the actual, correct response (x-axis). **(B)** An example of plots of adverse event prediction for the drug dasatinib. The predicted (blue markers) and actual proportions (orange markers) of individuals that experience adverse events related to a particular organ or body tissue (represented on the x-axis). The line connecting the marker represents the observed error between the predicted proportion of individuals that would experience adverse events against the actual proportion reported in clinical trials. Each prediction is obtained using a model that was trained without using the corresponding (held out) observations reported in breast cancer clinical trials that treated patients with dasatinib. **(C)** Box plot displaying the typical values of the reported adverse event affecting a particular tissue for the drug dasatinib and the predicted response, and any possible outliers. The central mark indicates the median, and the bottom and top edges of the box are the 25th and 75th percentiles, respectively. The whiskers extend from the boxes to the most extreme data points that are not considered outliers, whereas outliers are shown individually using the "+" symbol.

Our model accurately predicted the healthy tissues at risk of adverse events for each anticancer drug, based on the transcript levels of the targeted CSRs (R^2^ = 0.75; Fig 8A). Furthermore, the model estimated the percentage of individuals likely to suffer tissue-specific adverse events for each drug (Fig 8B and 8C; see Methods section). For instance, Fig 8B illustrates the model’s predictions for dasatinib (targeting ABL, SRC, EPH, PDGFR, and KIT) in predicting patient adverse reactions. S4 Fig demonstrates the model’s adverse event predictions for gemcitabine.

In conclusion, our machine learning approach accurately predicts which healthy tissues are prone to adverse toxic events from various anticancer drugs. This methodology holds potential in pre-emptively identifying adverse drug events during the stages of preclinical and clinical drug development.

## Discussion

Cell surface receptors (CSRs) are frequently overexpressed in various cancers, rendering them effective targets for small-molecule inhibitors and antibody therapies [56–59]. Our study reveals that CSR gene expression varies markedly across healthy tissues; a factor critical in predicting adverse reactions to anticancer drugs. Notably, some CSRs targeted in breast cancer treatment and abundantly expressed in tumours also show higher expression in certain healthy tissues, leading to significant drug-induced collateral damage [56–59].

Our study’s approach to predicting breast cancer subtypes through CSR transcription data offers a significant advancement in the understanding of breast cancer heterogeneity. By utilising a machine learning model, we successfully categorised tumours into PAM50 subtypes based solely on CSR expression levels, a method that could refine the current subtyping system predominantly reliant on genetic and phenotypic markers. This finding aligns with recent studies emphasising the role of transcriptomic profiling in improving cancer classification and prognosis [4,60,61], and by extension, how these profiles predicts the chemosensitivity of tumour to anticancer drugs [62].

Our analysis revealed that breast cancer cell lines with elevated CSR mRNA levels, corresponding to a specific drug target, tend to exhibit greater drug sensitivity. While prior research has linked tumour transcriptional profiles to drug responses [63–66], our focus on CSRs identified that the “ideal drug targets” are those highly expressed in breast cancer tumours but minimally in healthy tissues. This characteristic could enhance therapeutic efficacy and reduce adverse effects.

In exploring the dynamics of drug response in breast cancer, our findings resonate with recent studies that have similarly underscored the complexity of breast tumours at the transcriptome levels identifying specific genes that correlate with sensitivity or resistance to chemotherapy [67,68]. Other studies also support the link between the transcriptional heterogeneity in breast cancer within populations, including variation in genes targeted by microRNAs in modulating the chemosensitivity of tumours [69,70].

Our findings demonstrate that cell lines with higher expression levels of targeted CSRs tend to show increased sensitivity to specific drugs, a concept that resonates with recent research highlighting the utility of molecular profiling in enhancing drug efficacy [16,37,63,71]. This insight is particularly valuable in the context of personalised medicine, where understanding the molecular underpinnings of tumours can guide more effective and individualised treatment plans. Our study, therefore, contributes to the growing body of evidence suggesting that the integration of transcriptomic data can significantly improve therapeutic decision-making in breast cancer management.

Utilising clinical trial data, we found that treatments targeting CSRs with greater transcription levels in breast tumours than in healthy tissues reported fewer adverse events. By mapping CSR expression to specific tissue types and correlating this with adverse event data from clinical trials, we have provided a framework for predicting drug-induced toxicity, a key challenge in oncology treatment. This aligns with recent research correlating genetic variations in drug targets with side effects observed in specific organ systems [22] and emerging trends in the use of computational models for drug safety assessment [72–74]. The ability to predict adverse events accurately is paramount in drug development and patient care, potentially leading to safer treatment options and improved quality of life for patients undergoing cancer therapy.

Our findings carry substantial implications for choosing CSR drug targets to develop safer therapeutics. This method could also extend to identifying non-CSR drug targets that minimise toxicity in healthy tissues, allowing for higher drug dosages and safer antibody-drug conjugates with non-specific cytotoxins for disease treatment [59,75]. This strategy bypasses the biological significance of targeted CSRs in downstream signalling pathways, focusing instead on any CSR present in tumour cells but absent in healthy cells. Furthermore, such an approach promises fewer disease remissions and enhanced efficacy compared to current treatments, as non-specific cytotoxins would target tumour cells with a specific molecular phenotype more effectively [71,76–79].

In conclusion, our study highlights the differential CSR expression between breast tumours and healthy tissues, a key consideration for maximising therapeutic benefits while minimising off-target toxicities in CSR-targeted therapies. Additionally, our findings suggest the feasibility of using publicly available datasets to adopt a similar computational approach for non-CSR drug target selection.

## Supporting information

S1 Fig Comparative analysis of drug response significance across breast cancer subtypes by PAM50 classification. The colours show the degree of statistical significance (i.e., p values), with redder colours denoting smaller (more significant) p-values and greener colours denoting larger p-values. The marks are labelled with p-values.
(EPS)

S2 Fig Expression variability of clinical significance receptor genes across normal tissues. The heatmap of the log-2 fold-change values of the CSR transcripts of some anticancer drug targets utilised in the clinical trial data analysed.
(EPS)

S3 Fig Prevalence of adverse events in clinical trials involving filgrastim as documented by clinicaltrials.gov identifiers. Each bar shows the percentage of individuals that experienced the adverse events broken down by sub-adverse events for each clinical trial denoted by ClinicalTrials.gov. The colours show details about adverse events categories.
(EPS)

S4 Fig Comparison of predicted and actual adverse event frequencies for gemcitabine treatment. The predicted (blue markers) and actual proportions (orange markers) of individuals that experience adverse events related to a particular organ or body tissue (represented on the x-axis). The line connecting the marker represents the observed error between the predicted proportion of individuals that would experience adverse events against the actual proportion reported in clinical trials. Each prediction is obtained using a trained model without using the corresponding (held out) observations reported in breast cancer clinical trials that treated patients with gemcitabine.
(EPS)

S1 File Data of differentially expressed CSRs between breast cancer and all other healthy body tissues and organs: The spreadsheet contains the following results according to the sheet name. ***Up Genes—BRCA vs All Normal***; list of genes we found highly expressed in breast cancer than in any other healthy body tissue. ***Up Genes Counts- BRCA vs Normal***; the number of upregulated genes between breast cancer and each healthy tissue, and the number of downregulated genes between breast cancer and each healthy tissue.
(XLSX)

S2 File All differentially expressed CSRs between breast cancer and each healthy tissue. Each sheet is named according to the comparison for which the differential CSR expression results are represented. E.g., BRCA vs skin shows the results between breast tumour profile by the TCGA and all healthy tissues profile by the GTEx project.
(XLSX)

S3 File Differentially expressed CSRs between PAM50 subtypes of breast cancer. The sheets are named according to the comparison for which the differential expression CSRs results are represented. E.g., BRCA Basal-Vs-BRCA HER2 shows the results of Basal-like breast cancer subtype and HER2-positive breast cancer.
(XLSX)

S4 File Drug-response differences. The spreadsheet contains the following results according to the sheet name. ***Dose-Response of cell lines***; collated data of breast cancer cell lines profiled by the GDSC and the PAM50 subtype of each breast cancer cell line. ***Dose Response Anova Results***; comparison of dose-response between each PAM50 subtype of breast cancer. ***Anova Statistics***; ANOVA statistic for the comparison in the "Dose Response Anova Results" spreadsheet. ***CSR_based Response Comparison***; Dose-response comparison between the GDSC breast cancer cell line that we segregated into two groups, those that expressed higher amounts of a particular drug target and those that expressed lower amounts of a particular drug target.
(XLSX)

S5 File Spreadsheet showing the information that we collated from various results, including clinical trials information from www.clinicaltrials.gov of drug targets for drugs used applied in clinical trials that were retrieved from the Pharos database and Drug Gene Interaction database and the CSR transcript levels of the tissues in which are adverse events that are reported in the clinical trials occurred. (XLSX)
